# Health and Disease Imprinted in the Time Variability of the Human Microbiome

**DOI:** 10.1128/mSystems.00144-16

**Published:** 2017-03-21

**Authors:** Jose Manuel Martí, Daniel Martínez-Martínez, Teresa Rubio, César Gracia, Manuel Peña, Amparo Latorre, Andrés Moya, Carlos P. Garay

**Affiliations:** a Institute for Integrative Systems Biology (I2SysBio), Valencia, Spain; b Instituto de Física Corpuscular (IFIC), Valencia, Spain; c FISABIO, Valencia, Spain; d Cavanilles Institute of Biodiversity and Evolutionary Biology, Valencia, Spain; e CIBER en Epidemiología y Salud Pública (CIBEResp), Madrid, Spain; University of Chicago

**Keywords:** ecological modeling, metagenomics, microbiome, stability, systems biology

## Abstract

The animal microbiota (including the human microbiota) plays an important role in keeping the physiological status of the host healthy. Research seeks greater insight into whether changes in the composition and function of the microbiota are associated with disease. We analyzed published 16S rRNA and shotgun metagenomic sequencing (SMS) data pertaining to the gut microbiotas of 99 subjects monitored over time. Temporal fluctuations in the microbial composition revealed significant differences due to factors such as dietary changes, antibiotic intake, age, and disease. This article shows that a fluctuation scaling law can describe the temporal changes in the gut microbiota. This law estimates the temporal variability of the microbial population and quantitatively characterizes the path toward disease via a noise-induced phase transition. Estimation of the systemic parameters may be of clinical utility in follow-up studies and have more general applications in fields where it is important to know whether a given community is stable or not.

**IMPORTANCE** The human microbiota correlates closely with the health status of its host. This article analyzes the microbial composition of several subjects under different conditions over time spans that ranged from days to months. Using the Langevin equation as the basis of our mathematical framework to evaluate microbial temporal stability, we proved that stable microbiotas can be distinguished from unstable microbiotas. This initial step will help us to determine how temporal microbiota stability is related to a subject’s health status and to develop a more comprehensive framework that will provide greater insight into this complex system.

**Author Video**: An author video summary of this article is available.

## Abstract (precis)

The human microbiota correlates closely with the health status of its host. This article analyzes the microbial composition of several subjects under different conditions over time spans that ranged from days to months. Using the Langevin equation as the basis of our mathematical framework to evaluate microbial temporal stability, we proved that stable microbiotas can be distinguished from unstable microbiotas. This initial step will help us to determine how temporal microbiota stability is related to a subject’s health status and to develop a more comprehensive framework that will provide greater insight into this complex system.

## INTRODUCTION

The quest to understand the factors that influence human health and cause disease has always been one of the major driving forces of biological research. With growing evidence of the new “holobiont” and “hologenome” concepts (1, 2), research focuses not only on human physiology but also on the associated microbial population, although these concepts are still under debate (3). Research has revealed that the human microbiome is intimately linked to our physiology through the metabolism of bile acids (4), choline (5), and key metabolites such as short-chain fatty acids (6, 7), which are also involved in immune system maturation (8, 9). The human microbiota is plausibly related to diseases such as type 2 diabetes (10), cardiovascular disease (11), irritable bowel syndrome (IBS) (12), and Crohn’s disease (13) and some afflictions like obesity (14, 15) and malnutrition (16), as well as many other diseases (17). Current studies have revealed that gut microbes also influence brain function and behavior and are related to neurological disorders like Alzheimer’s disease through the gut-brain axis (18, 19). Recently, chronic fatigue syndrome (CFS), a subtle but devastating condition often cited as a psychosomatic disease, has been associated with a reduced diversity and altered composition of the gut microbiome (20).

This research area has progressed greatly thanks to high-throughput methods for microbial 16S rRNA gene sequencing and SMS (shotgun metagenomic sequencing), which reveal the composition of archaeal, bacterial, fungal, and viral communities located in and on the human body. Modern high-throughput sequencing and bioinformatics tools provide a powerful means of understanding how the human microbiome contributes to health and its potential as a target for therapeutic interventions (21). Research is under way to establish normal host-gut microbe interactions and understand how microbiota compositional changes can cause certain diseases (22–24).

Biology has recently acquired new technological and conceptual tools to investigate, model, and understand living organisms at a systems level, thanks to progress in quantitative techniques, large-scale measurement methods, and joint experimental and computational approaches. In particular, systems biology strives to reveal the general laws governing the complex behavior of microbial communities (25–27), including a proposal for universal dynamics (28). Microbiota can be approached in the light of ecological theory, which includes general principles like Taylor’s law (29, 30) relating the spatial or temporal variability of the population with its mean. This law, also known as the fluctuation scale law, is ubiquitous in the natural world and can be found in several systems such as random walks (31), stock markets (32, 33), tree (34) and animal (30, 35, 36) populations, gene expression (37), and the human genome (38). Taylor’s law has been applied to microbiotas spatially by Zhang et al. (39), with results showing that this population tends to be an aggregated one rather than having a random distribution. Despite its ubiquity, this law has only been tested in experimental settings (40, 41) and has never been applied in follow-up studies on microbiota, despite major efforts to infer the community structure from a dynamic point of view (42–44).

This paper presents the hallmarks of health status (healthy or diseased) in the macroscopic properties of the microbiota by studying its temporal variability. We analyzed over 40,000 time series of taxa from the gut microbiomes of 99 subjects obtained from publicly available high-throughput sequencing data related to different conditions, i.e., diseases, diets, trips, obesity, antibiotic therapy, and healthiness. On finding that all of the cases followed Taylor’s law, we used this empirical fact to model how the relative abundances of taxa evolved over time by using the Langevin equation, similarly to the approach applied by Blumm et al. (45). We used this mathematical framework to explore the temporal stability of the microbiotas under different conditions to understand how this is related to the health status of the subjects.

## RESULTS

**FIG 1:**
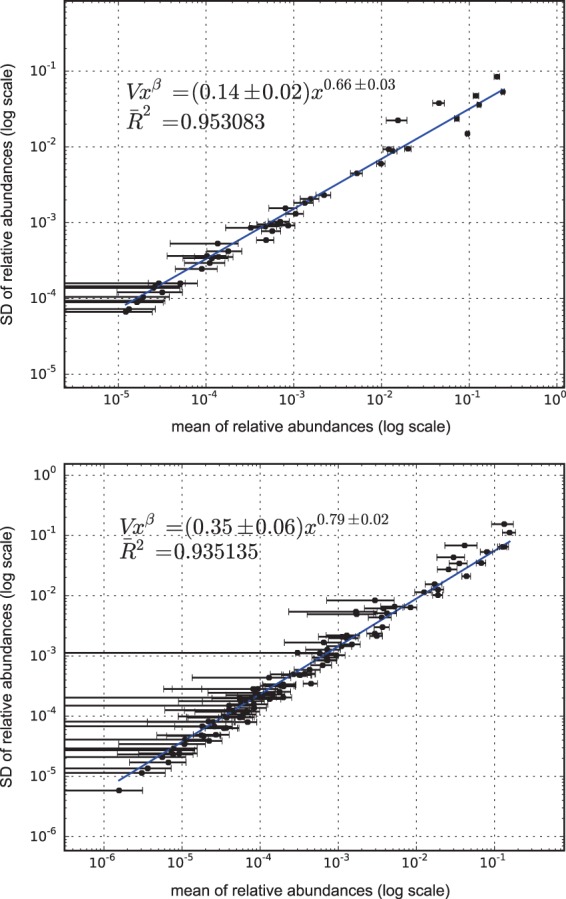
*x*-weighted power law fits of the standard deviations (SD) versus the mean values for each bacterial genus monitored over time. The fit is shown for samples from a healthy subject (top) and from a subject with a diagnosis of IBS (bottom) studied in our lab (12). Taylor’s power law seems to be ubiquitous, spanning 6 orders of magnitude. *V* corresponds to the *y* intercept, and β corresponds to the slope of the line. The error bars (mean axis) are the SEM.

Microbiome temporal variability was analyzed to extract the global properties of the system. As fluctuations in total counts are plagued by systematic errors, we worked on the temporal variability in the relative abundance of each taxon. Our first finding was, without exception, that changes in the relative abundances of taxa followed a ubiquitous pattern, known as the fluctuation scaling law (46) or Taylor’s power law (30). In other words, the microbiota of all of the taxa detected followed ${\text{σ}}_{i}=V\cdot x_{i}^{\text{β}}$, a power law dependence between mean relative abundance *x*_*i*_ and dispersion σ_*i*_. The law seems to be ubiquitous, even spanning 6 orders of magnitude in the observed relative abundances. As shown in Fig. 1, where *V* corresponds to the *y* intercept and β is the slope of the fit, the most abundant species were less volatile in relative terms than the less abundant ones. The fit to the power law was always robust (*R*^2^ > 0.88) and did not depend on microbiome condition. The power law (or scaling) index β and the variability *V* (hereafter Taylor’s parameters) appear to be correlated with community stability. Accordingly, we assume that Taylor’s parameters behave as proxies for stability. On the one hand, β is a scaling index that provides information about the statistical properties of the ecosystem. If it is 1/2, the system behaves like a Poisson distribution. If β is 1, the system behaves like an exponential distribution. Generally speaking, metagenomes undergo time course variations with β between these two universal classes. In our study, the fact that β was less than 1 indicates that the most abundant taxa in the microbial community were less susceptible to perturbations than the other taxa. On the other hand, the variability *V* is a direct estimator of the amplitude of fluctuations over time. *V* represents the maximum variability attainable by a hypothetically dominant genus (with relative abundance close to 1). It is an important parameter that characterizes the type of system. If *V* is small, the ranking is stable. For example, this would be the case for the number of diagnoses of a particular disease recorded in Medicare during a month (47). If *V* is large, as occurs for metagenomic samples, the ranking might be unstable, as it would be for the number of hourly page views of articles in Wikipedia (45, 46). Interestingly, the Taylor parameters were related to the health status of the host, which is the main finding of this study.

Taylor’s parameters describing the temporal variability of the gut microbiome in our sampled subjects are shown in Tables S1 to S4 in the supplemental material. Our results are indicative of ubiquitous behavior. First, the variability (corresponding to the maximum amplitude of fluctuations) was large, which suggests resiliency of the microbiota. Second, the scaling index was always smaller than 1, which means that the more abundant taxa were less volatile than the less abundant ones. In addition, Taylor’s parameters for the microbiome of healthy subjects in different studies (12, 48–53) were compatible with estimated errors. This enabled us to define an area in the Taylor parameter space that we called the healthy zone.

10.1128/mSystems.00144-16.6 TABLE S1  Taylor’s parameters of subjects with either animal-based (A) or plant-based (P) diets (52). Previous to the diet, the population sampled was described by *V̄* = 0.09 ± 0.05 and β̄ = 0.77 ± 0.04. Download TABLE S1, PDF file, 0.03 MB. Copyright © 2017 Martí et al. 2017 Martí et al. This content is distributed under the terms of the Creative Commons Attribution 4.0 International license.

10.1128/mSystems.00144-16.7 TABLE S2  Taylor’s parameters for subjects taking antibiotics (Ab) in the antibiotic study (49), persons with a diagnosis of IBS in the IBS study (12) and for special intervals concerning gut microbiota in the host lifestyle study (HLS) (53). Prior to antibiotic intake, the population sampled in the antibiotic study (49) was described by *V̄* = 0.12 ± 0.05 and β̄ = 0.75 ± 0.04. Healthy subjects sampled in the IBS study (12) were characterized by *V̄* = 0.135 ± 0.010 and β̄ = 0.692 ± 0.024. The healthy and quotidian periods in the host lifestyle study (53) are characterized by *V̄* = 0.25 ± 0.09 and β̄ = 0.777 ± 0.025. Download TABLE S2, PDF file, 0.03 MB. Copyright © 2017 Martí et al. 2017 Martí et al. This content is distributed under the terms of the Creative Commons Attribution 4.0 International license.

10.1128/mSystems.00144-16.8 TABLE S3  Taylor’s parameters for the healthy-subject (DH) and kwashiorkor (DK) parts of the discordant-twin studies (51). The population of healthy twins is characterized by *V̄* = 0.25 ± 0.10 and β̄ = 0.863 ± 0.028. Download TABLE S3, PDF file, 0.03 MB. Copyright © 2017 Martí et al. 2017 Martí et al. This content is distributed under the terms of the Creative Commons Attribution 4.0 International license.

10.1128/mSystems.00144-16.9 TABLE S4  Taylor’s parameters for subjects with various degrees of overweight and obesity (50). Healthy people in this study, who were not obese, are characterized by *V̄* = 0.19 ± 0.06 and β̄ = 0.806 ± 0.034. Download TABLE S4, PDF file, 0.03 MB. Copyright © 2017 Martí et al. 2017 Martí et al. This content is distributed under the terms of the Creative Commons Attribution 4.0 International license.

**FIG 2:**
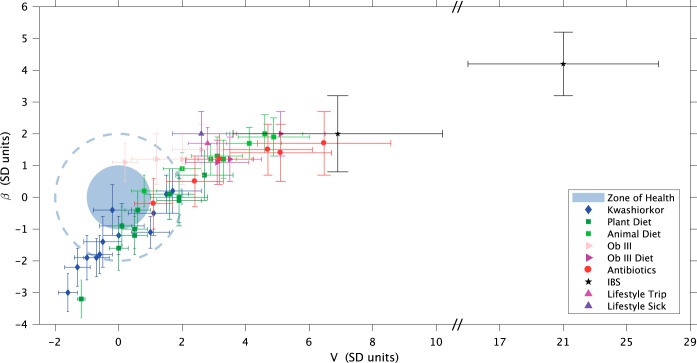
Taylor’s law parameter space. All of the data studied in this work are compiled here. The large light-blue circle corresponds to the 68% CL region of healthy subjects in the Taylor parameter space, while the dashed circumference around it delimits the 98% CL region. Points with errors place the gut microbiome in the Taylor parameter space for each subject whose microbiota was compromised. It should be noted that the parameters were standardized (standard deviation units) to the healthy group for every single study independently for purposes of demonstration and comparison.

To jointly visualize and compare the results of subjects from the above-mentioned studies (12, 48–53), their Taylor parameters were standardized, with standardization meaning that the mean value was subtracted from each parameter and the result was divided by the standard deviation of the group of healthy subjects for every study independently. Because of the different systematics in each study, we defined a healthy region for each of them, standardized to a mean of 0 and a variance of 1, then we computed the mean and variance of the “unhealthy” with this standardization (for details of the procedure, see the standardization subsection in Materials and Methods). Therefore, different studies were isolated so that the subjects in any study did not affect the results for the “unhealthy” subjects in the other studies. We think this statistical approach was safer, as we avoided combining data with very different systematic errors. The healthy zone and the standardized Taylor parameters for the temporal variability of the gut microbiome in subjects whose gut microbiota was compromised (i.e., they had IBS, kwashiorkor, an altered diet, antibiotic intake, or a *Salmonella* infection or had gone on a trip abroad) are shown in Fig. 2. The variability in children with kwashiorkor was smaller than that of their healthy twins. A meat/fish-based diet significantly increased variability compared to a plant-based diet. All other cases presented increased variability, which was particularly severe and statistically significant at an over 95% confidence level (CL), for grade III obese patients on a diet, subjects taking antibiotics, the subject who had a *Salmonella* infection, the subject who had traveled abroad, and the patients with a diagnosis of IBS. One global property that emerges from these comprehensive data is that the Taylor parameters characterized the statistical behavior of microbiome changes. Furthermore, we verified that our conclusions were robust to systematic errors resulting from taxonomic assignment (see taxon level selection in Materials and Methods).

**FIG 3:**
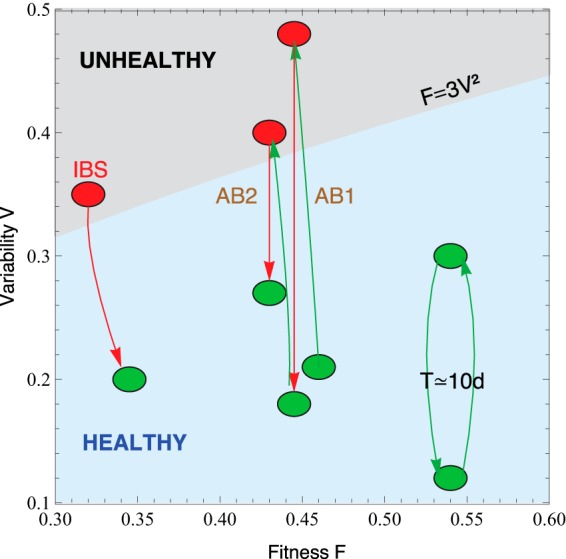
Microbiota states can be placed in the phase space *F-V*. The light-blue-shaded region corresponds to the stable phase, while the gray-shaded region is the unstable phase (the phase transition line is calculated for α = β = 0.75). We placed healthy subjects (green) and subjects whose gut microbiota is threatened (antibiotics, IBS) in the phase space fitness-variability. The gut microbiotas of healthy subjects over a long time span show a quasiperiodic variability (the central period is 10 days). We show that taking antibiotics (AB1 and AB2 correspond to the first and second treatments, respectively) induces a phase transition in the gut microbiota that has an impact on future changes. We also show a patient with a diagnosis of IBS transitioning from the unstable to the stable phase.

Taylor’s power law has been explained in terms of various effects, though without a general consensus. It has its origin in mathematical convergence, which is similar to the central limit theorem, and thus, virtually any statistical model designed to produce a Taylor law converges to a Tweedie distribution (54), providing a mechanistic explanation based on the statistical theory of errors (55–57). To reveal the generic mechanisms that drive different scenarios in the β-*V* space, we modeled the system by assuming that taxon relative abundance followed a Langevin equation with, on the one hand, a deterministic term that captured the fitness of each taxon and, on the other hand, a randomness term associated with Gaussian random noise (45). Both terms were modeled by power laws, with coefficients that can be interpreted as taxon fitness *F*_*i*_ and variability *V* (see model in Materials and Methods). *F*_*i*_ captures the time scale that the system needs to reach equilibrium (the size of *V* may or may not allow equilibrium to be reached). *F*_*i*_ has dimensions of 1/time and roughly corresponds to the half-life of the system when it is decaying to a stable state. In fact, it is exactly the half-life if β is 1 and *V* is negligible. In this model, when *V* is sufficiently low, abundances are stable in time. Differences in *V* can induce a noise-induced phase transition in the relative abundances of taxa. The Fokker-Planck equation governs the temporal changes in the likelihood that a given taxon has an abundance *x*_*i*_, given its fitness. The results of solving this equation show that stability is best captured by a phase space determined by the fitness (*F*) and the amplitude of fluctuations (*V*) (Fig. 3).

The model predicted two phases for the gut microbiome, a stable phase with large variability that enabled some changes in the relative abundances of taxa and an unstable phase with even larger variability, above the phase transition, where the order of abundant taxa varies significantly over time. The phase transition is continuous (of second order), as is the crossing of the boundary. The state variable is the composition. Any disturbance modifies the composition of the microbiota, with different compositions encoding different *F* and *V* values. We have shown that effective perturbations significantly change *V* and lead the microbiota to a transition from the ordered phase to the noise-induced one. Our model can be solved analytically, which allows for a simple understanding of the different regimes and, in particular, to calculate the formula of the transition region. The order parameter is the composition *x*_*M*_ that maximizes the probability distribution; 0 < *x*_*M*_ < 1 defines the ordered phase, while *x*_*M*_ > 1 defines the disordered phase. If *V* is sufficiently smaller than *F*, likelihood peaks in the physical region (relative compositions larger than 0 and smaller than 1); i.e., there is a best-composition solution of the differential equation, which is the ordered solution. Conversely, if *V* is sufficiently larger than *F*, the likelihood peaks outside the physical region; i.e., the best-composition solution of the differential equation is at the boundaries (either 0 or 1) and all physical solutions have comparable likelihoods, i.e., the noise-induced phase. The microbiomes of healthy subjects were found to be in the stable phase, while the microbiomes of several other subjects were in the unstable phase. In particular, subjects taking antibiotics and patient P2 (with a diagnosis of IBS) had the most severe symptoms. In this phase diagram (Fig. 3), each microbiota state is represented by a point at its measured *V* and inferred *F*. The model predicted high average fitness for all taxa; i.e., taxa were narrowly distributed in *F*. The fitness parameter was chosen with different values as a demonstration. Fitness was larger for the healthiest subjects and smaller for the patients with a diagnosis of IBS.

### Rank stability of the taxa.

**FIG 4:**
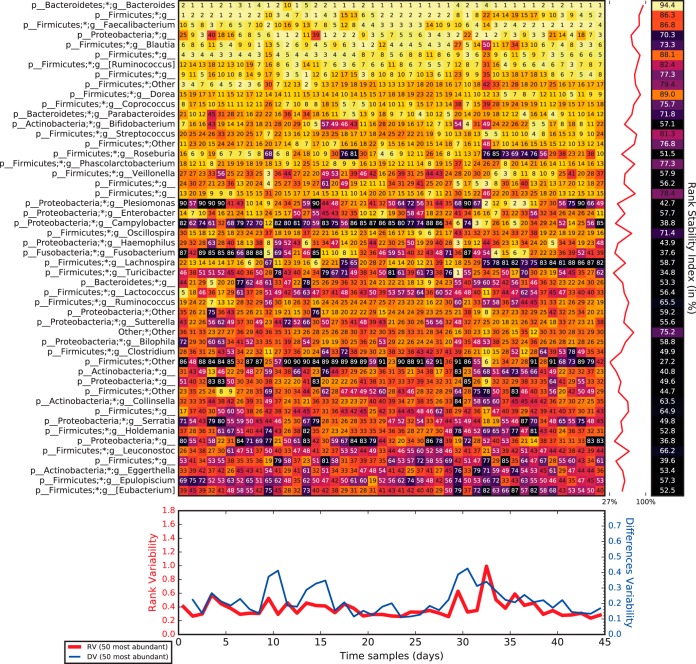
Rank variation over time of the 50 most dominant elements (taxa) and their calculated RSI, RV, and DV, as detailed in rank stability and variability in Materials and Methods, for a special period (days 72 to 122, traveling abroad) belonging to subject A in the host lifestyle study (53).

**FIG 5:**
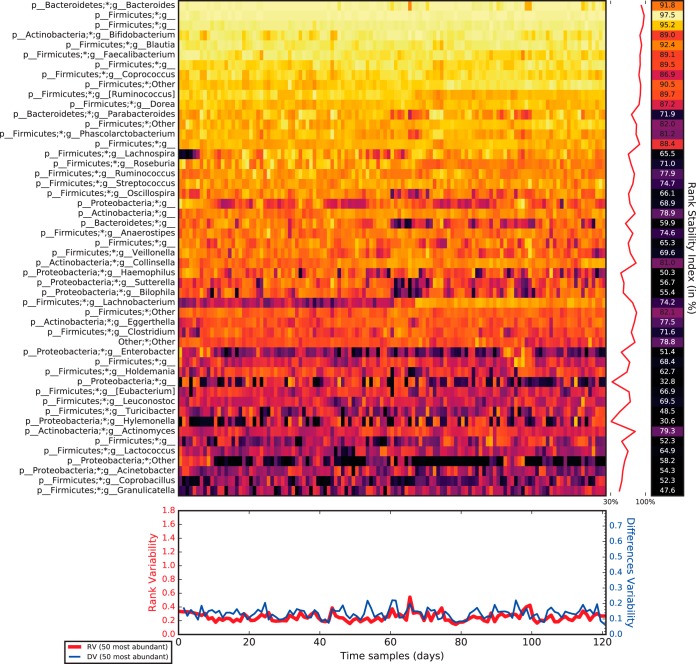
Rank variation over time of the 50 most dominant elements (taxa) and their calculated RSI, RV, and DV, as detailed in rank stability and variability in Materials and Methods, for an ordinary period (days 123 to 256, after the trip) belonging to subject A in the host lifestyle study (53).

The rank dynamics and stability plots in Fig. 4 and 5 show the variations in rank over time for the most dominant taxa and their calculated rank stability index (RSI; as discussed in Materials and Methods) for the gut microbiome taxa of a healthy subject, namely, subject A in the host lifestyle study (53). Figure 4 covers the period when the subject was traveling abroad, and Fig. 5 covers the subsequent period. The taxa are listed according to their accumulated frequency over the time series, with the *y* axis being the overall dominant axis for each sample set. Generally speaking, we observed that the most dominant taxa had the highest rank stability.

For the trip abroad in Fig. 4, beyond the differences in the dominance of particular taxa, we observed that the most dominant ones were also the most rank stable ones. Moreover, the medium-ranked taxa were quite rank unstable, mostly because of transient (often one or two consecutive samples), albeit dramatic, declines in their relative abundance, which usually occurred more than twice during their time series.

Nevertheless, in the case of the next period (Fig. 5), the one subsequent to the trip, some taxa showed higher stability than other more dominant taxa, forming “rank stability islands” for medium-ranked taxa, and displaying a moderately stable index (RSI roughly over 70%). In particular, this was the case for the genera *Actinomyces*, *Leuconostoc*, *Lachnobacterium*, *Eggerthella*, *Clostridium*, and *Collinsella*. For these genera, both the overall rank and the RSI were clearly lower during the trip (RSI under 70%). *Actinomyces* and *Lachnobacterium* are not shown in Fig. 4 because they sank to positions 56 and 77, respectively. In contrast, *Leuconostoc* was the least sensitive to the lifestyle change. Interestingly *Lachnobacterium* showed anticorrelation over time compared to the vast majority of the taxa classified in this study.

We also found those “rank stability islands” for medium-ranked taxa in the other periods belonging to subject A in the host lifestyle study (53) (see Fig. S1 and S2 for the corresponding rank plots). See Table S5 for details of the rank and RSI of the above-mentioned taxa over the different periods considered.

10.1128/mSystems.00144-16.1 FIG S1  Rank variation over time of the 50 most dominant elements (taxa) and their calculated RSI, RV, and DV, as detailed in rank stability and variability in Materials and Methods, for an ordinary period (days 0 to 70, before the trip) belonging to subject A in the host lifestyle study (53). Download FIG S1, EPS file, 1.1 MB. Copyright © 2017 Martí et al. 2017 Martí et al. This content is distributed under the terms of the Creative Commons Attribution 4.0 International license.

10.1128/mSystems.00144-16.2 FIG S2  Rank variation over time of the 50 most dominant elements (taxa) and their calculated RSI, RV, and DV, as detailed in rank stability and variability in Materials and Methods, for an ordinary period (days 257 to 364, further after the trip) belonging to subject A in the host lifestyle study (53). Download FIG S2, EPS file, 1.2 MB. Copyright © 2017 Martí et al. 2017 Martí et al. This content is distributed under the terms of the Creative Commons Attribution 4.0 International license.

10.1128/mSystems.00144-16.10 TABLE S5  Rank and RSI, as discussed in Materials and Methods, over different periods for the taxa listed as rank stability islands regarding the gut microbiome of subject A in the host lifestyle study (53). Download TABLE S5, PDF file, 0.04 MB. Copyright © 2017 Martí et al. 2017 Martí et al. This content is distributed under the terms of the Creative Commons Attribution 4.0 International license.

### Time dependence of model parameters.

**FIG 6:**
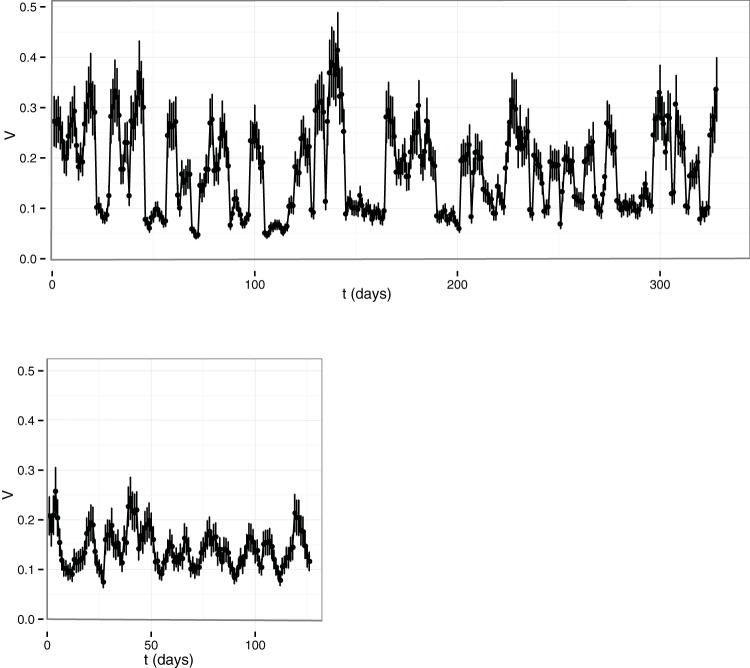
*V* of the two subjects in the study of Caporaso et al. (48) as a function of time. Samples of the gut microbiomes of a male (top) and a female (bottom) are shown.

Finally, we studied the time dependence of variability *V* and the power law index β (see model in Materials and Methods) by using a sliding-window approach. The total number of time points was divided into subsets of five points, where the following subset was defined by adding the next sampling time and eliminating the earliest one. Both parameters were calculated for each subset against the average time lapse. Figure 6 shows variability *V* as a function of time for the two subjects in the study of Caporaso et al. (48) corresponding to the gut microbiota of a male (upper plot) and a female (lower plot). Both samples showed changes in *V* with quasiperiodic behavior peaking at about 10 days. Variability grew more for the gut microbiota of the male and shared a minimal value of around 0.1 with the gut microbiota of the female.

**FIG 7:**
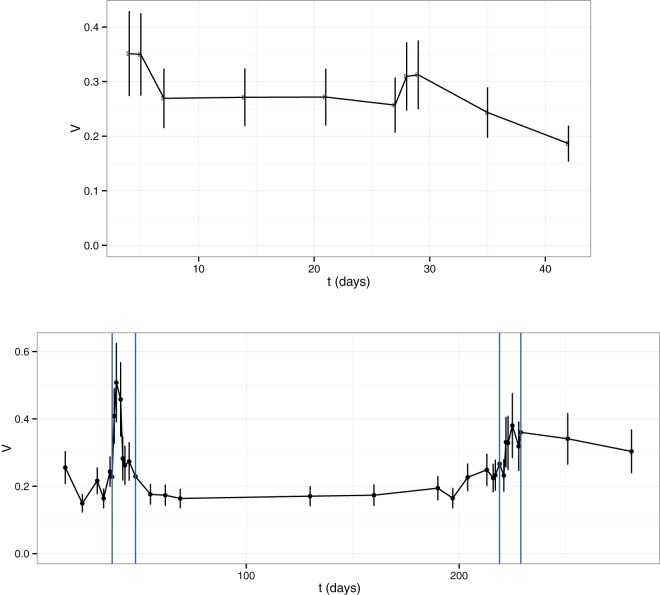
*V* as a function of time for patient P2 in the IBS study (12) (top) and patient D in the antibiotic study (47) (bottom). The blue vertical lines in the lower plot show the periods of antibiotic treatment.

Figure 7 shows time course changes in *V* for patient P2 in the IBS study (13) (upper plot) and patient D in the antibiotic study (49) (lower plot). The variability of the gut microbiota of P2 decreased from over 0.3 to below 0.2, showing a slow tendency to increase the order of the system. Antibiotic intake led to a quick increase in variability that lasted a few days until *V* recovered a low level. The second antibiotic treatment showed some memory traits (smaller increase in variability) with slower recovery.

## DISCUSSION

One of the highlights of this study is that it shows, independently of its condition, that the microbiota follows Taylor’s law. We have seen that, in each case, the value of the scaling index is always less than unity (using the standard deviation as the dispersion measurement), which provides us with information about the community structure. This means that, in relative terms, the most abundant elements in the population are less volatile to perturbations than the less abundant ones. The explanation for this universal pattern is not clear, although some hypotheses have been tested in other studies, such as the presence of negative interactions in the population (58), and a demonstration that this may depend on reproductive correlation (59). Nevertheless, none of these explanations is sufficient when it comes to the microbiota, as the term reproduction is diffuse and the interactions between its components are not only based on competition (60–62). Moreover, even such a negative interaction may not effectively yield values of less than unity when referring to a bacterial species (41). Nonetheless, the values obtained in all cases were very similar to each other, which may suggest that the community structure is preserved throughout the different scenarios studied here.

The second parameter provides information about noise and can be directly linked to the variability or fluctuation amplitude of the population over time. It is a direct estimator of the stability of the system under study. As we have shown above, the healthy subset in each study has less variability than the nonhealthy subset when adult subjects are considered. Interestingly, the variability parameter was higher in the healthy subset in the study of discordant twins suffering from kwashiorkor disease (51). In this respect, research has shown that the infant microbiota needs to develop toward a definite, adult state (63). This implies that temporal variability is greater in children than in healthy adults, who should be temporally stable. Thus, our results could indicate that this variability is necessary to reach that adult state. Furthermore, as we wanted to see how this variability shifted over time, we calculated the changes in this parameter for the samples that had enough sampling coverage. As shown in Fig. 6, the variability of the microbiota fluctuated over time. Interestingly Fig. 7 shows how this parameter reflected the two antibiotic intakes by one of the patients in the study by Dethlefsen and Relman (49), particularly the apparent resilience of the microbiota due to the reduced increase in variability during the second antibiotic intake.

The primary hypothesis of this work is that, in adults, having a healthy microbiota means that the microbial population is stable over time. This stability means that the microbiota does not shift and become susceptible to external or internal perturbations causing dysbiosis. To use the valuable information provided by the empirical law of Taylor’s work, here we have proposed the use of Langevin’s equation to model how stability ranking changed over time. While the system noise component can be directly measured as its variability, the other main term needs to be inferred from the model. This term, which we have named “fitness,” enables the system to remain stable when confronted with potential perturbations. In ecological terms, this could represent the nature of the interactions present among bacteria, between bacteria and other minority populations such as fungi or archaea, and between bacteria and the viral component of the microbiota and interactions between the host and the whole microbiota. As this is a first step in modeling the temporal stability of the microbiota and given its complex nature, we calculated fitness by using the fluctuation dissipation theorem as a first approximation (64). Thus, further work is required to model the fitness of the microbiota to provide a more accurate model with greater predictive power.

By solving Langevin’s differential equation, we obtained a phase diagram in which each microbiota sample can be placed by its fitness and variability into one of two phases, according to the stability ranking of the system. As shown by the phase space in Fig. 3, three different conditions can occur. The first is a healthy microbiota with some fluctuations, as shown by one of the subjects in the study of Caporaso et al. (48). As this case would have good fitness, its temporal variability would not place the microbiota in the unstable phase of the diagram. Second, we have a subject from the study of Dethlefsen and Relman (49) whose microbiota was perturbed twice by antibiotic intake, undergoing sufficient change to lose its stability and hence be placed in the unstable part. In this location, it is more sensitive to potential perturbations such as, for example, opportunistic infections. In the third and last condition, the subject was already in the unstable phase because of a health issue, i.e., IBS. This can be observed in one of the patients in the study of Durbán et al. (12). In addition, it was shown that this subject’s health status improved during the experiment, implying that his/her microbiota also recovered stability. Interestingly, in the study of David et al. (53), the subject who had a *Salmonella* infection during the experiment underwent a significant shift in variability with eventual recovery from the perturbed state (see Fig. S3).

10.1128/mSystems.00144-16.3 FIG S3  Taylor’s law parameter space for intervals concerning gut microbiota in the host lifestyle study (53). We observe that subject B, who suffered a *Salmonella* infection during the experiment, had a relevant shift in the parameters from “_before” to “_infection” and a final recovery from the perturbed state to “_afterinfec,” which lies in the parameter area compatible with the healthy and stable intervals (see Table S2). Subject A also had a shift in variability from “_before” to “_abroad” and back to “_returned,” also in the proximity zone of healthy and stable periods. Download FIG S3, EPS file, 0.4 MB. Copyright © 2017 Martí et al. 2017 Martí et al. This content is distributed under the terms of the Creative Commons Attribution 4.0 International license.

Specifically, in the host lifestyle study (53), the presence of “rank stability islands” among medium-ranked taxa is an interesting feature revealed by the analysis of rank stability in different time periods in subject A. Interestingly, this stability was compromised when the period was not an ordinary one, suggesting that those taxa were sensitive to lifestyle changes. Among the genera identified as “rank stability islands,” *Lachnobacterium* and *Clostridium* were catalogued as genera predictive of dysbiosis in the work of Larsen and Dai (65), which analyzed the same data set (53). Furthermore, research has recently confirmed a clear relationship between *Actinomyces* and conventional adenoma (66), one of the two main precursors of colorectal cancer. Finally, *Eggerthella* is an opportunistic pathogen often associated with serious gastrointestinal pathology (67).

One might question the role of these taxa as key players in the phase transition of the microbiota and wonder whether they are more susceptible to perturbations than the most abundant taxa. The types of interactions that could sustain this particular behavior are unclear, as these nonabundant taxa are usually excluded from dynamic studies to obtain a community matrix. Further experiments and data analysis are needed to clarify whether “rank stability islands” are a widespread feature of microbiotas and whether they appear at lower taxonomic levels too.

Notwithstanding the above, we should be aware that the hypothesis is too simplistic to directly apply to reality. Indeed, the situation is more complex than the idea that healthy people can be distinguished from nonhealthy people in solely compositional terms, as highlighted by Moya and Ferrer in their recent review (17). There are several feasible scenarios in which we can consider the microbiota to be stable, irrespective of its compositional shifts over time. For example, it may depend on the ability of the microbiota to recover its initial composition (resilience) or its ability to recover its original function despite its composition (functional redundancy). What we have shown in this work could be explained as the transition of a stable microbiota into a state of dysbiosis.

This is a first step toward understanding microbiota stability, although the model presents some limitations and thus further research is required. From a biological perspective, many questions arise from this work. We have observed the same pattern in Taylor’s parameters under all of the conditions studied, but a pertinent question is whether this is really a universal feature in the hugely diverse microbial niches. Furthermore, another relevant question relates to mechanisms involved in the maintenance of population structure. Undoubtedly, the nature of the interactions between community components has a great bearing on this issue, and this is related to community fitness, as mentioned above. How we should address community fitness remains unclear, but studies like the one by Tikhonov (68) could point us in the right direction and help us to unravel the complexity of the microbiota and its relationship to host health.

## MATERIALS AND METHODS

### Model.

We modeled microbial abundances over time along the lines of Blumm et al. (45). The dynamics of taxon relative abundances was described by the Langevin equation, ${\overset{˙}{x}}_{i}=F_{i}\;\cdot \;x_{i}^{\alpha }+V\;\cdot \;x_{i}^{\beta }\xi_{i}(t)-\phi (t)\;\cdot \;x_{i},$ where *F*_*i*_ captured the fitness of the taxon *i*, *V* corresponded to the noise amplitude, and ξ_*i*_(*t*) was a Gaussian random noise with a mean of zero, 〈ξ_*i*_(*t*)〉 = 0, and variance uncorrelated in time, 〈ξ_*i*_(*t*)ξ_*i*_(*t*′)〉 = δ(*t*′ − *t*). The function ϕ(*t*) ensured normalization at all times, ∑*x*_*i*_(*t*) = 1, and corresponded to $\phi (t)=\Sigma F_{i}x_{i}^{\alpha }+\Sigma Vx_{i}^{\beta }\xi_{i}(t)$. The time course changes in the probability that a taxon *i* has a relative abundance *x*_*i*_(*t*), *P*(*x*_*i*_,*t*), was determined by the Fokker-Planck equation:

$$\frac{\partial P}{\partial t}=-\frac{\partial }{\partial x_{i}}\left[(F_{i}\;\cdot \;x_{i}^{\alpha }-\phi (t)\;\cdot \;x_{i})\;\cdot \;P\right]+\frac{1}{2}\frac{\partial^{2}}{\partial x_{i}^{2}}(V^{2}\;\cdot \;x_{i}^{2\beta }\;\cdot \;P)$$

The microbiota evolved toward a steady state with a time-independent probability depending on the values of α, β, *F*_*i*_, and *V*. For α < 1 (otherwise, systems are always unstable), the steady-state probability was localized in a region around a preferred value or broadly distributed over a wide range, depending on whether fitness *F*_*i*_ dominated or was overwhelmed by the noise amplitude *V*. The steady-state solution of the Fokker-Planck equation was given by:

$$P_{0}\left(x_{i}\right)=\;C_{ne}(\alpha ,\beta ,F_{i},V)\;\cdot \;x_{i}^{-2\beta }\;\cdot \;\exp\left[\frac{2F_{i}}{V^{2}}\frac{x_{i}^{1+\alpha -2\beta }}{1+\alpha -2\beta }-\frac{\phi_{0}}{V^{2}}\frac{x_{i}^{2-2\beta }}{1-\beta }\right]$$

if 2β ≠ 1 + α and

$$P_{0}\left(x_{i}\right)=\;C_{e}(\alpha ,\beta ,F_{i},V)\;\cdot \;x_{i}^{\frac{2F_{i}}{V^{2}}-2\beta }\;\cdot \;\exp\left[\frac{\phi_{0}}{V^{2}}\frac{x_{i}^{2-2\beta }}{1-\beta }\right]$$

if 2β = 1 + α, where

$$\phi_{0}={\left(\;\sum_{i}\;{F_{i}}^{\frac{1}{1-\alpha }}\right)}^{1-\alpha }$$

and *C*_*ne*_ and *C*_*e*_ are integrals solved numerically for the parameters of interest. The ordered phase occurred when the solution had a maximum in the physical interval (0 < *x*_*i*_ < 1). For larger *V* values, the transition to a disordered phase happened when the maximum shifted to the unphysical region, *x*_*i*_ < 0, which set the phase transition region *V*(α, β, *F*_*i*_). The phase transition region was calculated analytically in particular cases, $F_{i}^{2}=4\beta \phi_{0}V^{2}$ if β = α ≠ 1 and $F_{i}=\beta V^{2}$ if 2β = 1 + α, where the first case simplified to *F* = 3*V*^2^ if β = 0.75 and the fitness of this taxon dominated in ϕ_0_. In many physical systems (Brownian motion is the classic example [69]), the two terms of the Langevin equation are related. The fluctuation dissipation theorem states a general relationship between the response to an external disturbance and the internal fluctuations of the system (64). The theorem can be used as the basic formula to derive fitness from the analysis of fluctuations in the microbiota, assuming that it is in equilibrium (the ordered phase).

### Standardization.

To properly show all of the studies under common axes, we decided to standardize the Taylor parameters by using the group of healthy subjects for every single study independently. By this approach, all of the studies can be visualized in a shared plot with units of Taylor-parameter standard deviation on their axes.

For a Taylor parameter, e.g., *V*, the estimate of the mean (*V̂*) of the healthy subpopulation, composed of *h* subjects, is

$$\hat{V}=\frac{1}{W_{1}}\sum_{i=1}^{h}V_{i}\omega_{i}=\sum_{i=1}^{h}V_{i}\omega_{i}$$

as $W_{1}=\Sigma_{i}^{h}\omega_{i}=1$, since ω_*i*_ are normalized weights calculated as

$$\omega_{i}=\frac{\frac{1}{\sigma_{V_{i}}^{2}}}{\sum_{i}^{h}\frac{1}{\sigma_{V_{i}}^{2}}}$$

where σ_*Vi*_ is an estimate of the uncertainty in *V*_*i*_ obtained together with *V*_*i*_ from the *x*-weighted power law fit for healthy subjects.

Likewise, the estimation of the standard deviation for the healthy population (σ^V) is

$${\hat{\sigma }}_{V}=\sqrt{\frac{1}{W_{1}-\frac{W_{2}}{W_{1}}}\sum_{i=1}^{h}\left[\omega_{i}{\left(V_{i}-\hat{V}\right)}^{2}\right]}$$

where $W_{2}=\Sigma_{i}^{h}\omega_{i}^{2}$, which finally yields

$${\hat{\sigma }}_{V}=\sqrt{\frac{1}{1-\sum_{i}^{h}\omega_{1}^{2}}\sum_{i=1}^{h}\left[\omega_{i}{\left(V_{i}-\hat{V}\right)}^{2}\right]}$$

### Selection and methods.

The bacterial and archaeal taxonomic assignments were obtained by analyzing 16S rRNA sequences, which were clustered into operational taxonomic units (OTUs) with 97% sequence identity by QIIME (70). SMS data (51) were analyzed and assigned at the strain level by the Livermore metagenomic analysis toolkit (LMAT) (71), according to their default quality threshold. The genus, with the best balance between error assignment and the number of taxa, was chosen as our reference taxonomic level. We verified that our conclusions were not significantly affected by selecting the family or species as the reference taxonomic level (see Fig. S4).

10.1128/mSystems.00144-16.4 FIG S4  Overview of the comparison of different approaches based on adjacent taxonomic levels using plots in the Taylor parameter space. The former row of subfigures is for 16S rRNA, where the levels are family (blue circles) versus genus (purple triangles), whereas the latter row of subfigures is for SMS, where the levels are genus (blue circles) versus species (purple triangles). The left column shows the raw results, and the right column plots the standardized results (see standardization in Materials and Methods). Download FIG S4, EPS file, 0.4 MB. Copyright © 2017 Martí et al. 2017 Martí et al. This content is distributed under the terms of the Creative Commons Attribution 4.0 International license.

### Sample selection.

We chose studies about relevant pathologies containing metagenomic sequencing time data series of bacterial populations from humans in various healthy and nonhealthy states. Only subjects for whom three or more time points of data were available in databases were selected. The study by Caporaso et al. (48) was selected because it featured two healthy subjects measured over a very long time span, with almost daily sampling. The study of Faith et al. (50) was selected because of the body mass index (BMI) differences among the subjects. Moreover, some of them were on diets, which could be treated as system perturbations. Only those subjects whose BMI was normal or who were overweight were considered healthy. The study by Smith et al. (51) was selected for both the age of the patients and the rare disease. Regarding kwashiorkor, we considered only the discordant twins and deemed subjects unaffected by kwashiorkor as being healthy in each pair of patients. The study of David et al. (52) was selected for its differential diets. The healthy period was considered to be the initial samples of each subject before starting the diet, while the remaining time points were considered perturbations. The study of Dethlefsen and Relman (49) was selected because of the interesting treatment of two intakes of the same antibiotic by three different subjects. The healthy period was considered to correspond only to those times before any antibiotic treatment, whereas the periods during and after antibiotic intake were considered perturbations. The work of David et al. (53) was selected because of the comprehensive longitudinal data that it provides plus its complete metadata and the interesting events experienced by both subjects (an infection and a trip abroad). The healthy period was taken from time points before and after each event. Finally, we also considered a study carried out by Durbán et al. (12) in which the healthy subjects were considered those who did not suffer from IBS, while the patients who had this disease were taken as perturbations.

The metadata of each study are provided in Tables S1 to S4. The studies all used 16S rRNA gene sequencing, except for the study of the discordant kwashiorkor twins (51), in which both SMS and 16S rRNA data were used. In the latter case, we chose to work with SMS data to show that our method is valid, regardless of the source of taxonomic information. Each of the data sets was treated as follows.

### 16S rRNA sequence processing.

Reads from the selected studies were first quality filtered with the FastX toolkit (72), allowing only those reads scoring over 25 for quality in 75% of the complete sequence. 16S rRNA reads with 97% nucleotide sequence identity were then clustered into OTUs with the QIIME software package (70) (version 1.8). We used an open reference OTU picking workflow in all cases. The clustering method used was UCLUST, and the OTUs were matched against the Silva database (73) (version 111, July 2012) and were taxonomically assigned by a UCLUST-based consensus taxonomy assigner. The parameters used in this step were a similarity of 0.97, a prefilter percent identity of 0.6, 20 maximum accepts, and 500 maximum rejects.

### Metagenomic sequence processing.

Shotgun metagenomic sequences were analyzed with the LMAT software package (71) (version 1.2.4, with the February 2015 release of the LMAT Grand database). LMAT was run with a Bull shared-memory node belonging to the team’s high-performance computing cluster. It was equipped with 32 cores (64 threads available with Intel hyperthreading technology) since it had two Haswell-based Xeons (22 nm technology), E5-2698v3 2.3-GHz processors, sharing half a tebibyte of DRAM (dynamic random access memory). This node is also provided with a PCIe solid-state drive (SSD) card as NVRAM (nonvolatile random access memory) and the Micron P420m HHHL (half-height half-length) with 1.4 terabytes and 750,000 reading IOPS (input-output operations per second) of 4 kilobytes, achieving 3.3 GB/s. The computing node was supplied with a RAID-0 (striping) scratch disk area. We used the Grand database (74), released in February 2015, provided by the LMAT team, where Grand refers to a huge database that contains k-mers from all of the viral, prokaryotic, fungal, and protist genomes present in the NCBI database, the human reference genome (hg19), GenBank Human, and the 1000 Human Genomes Project (this represents about 31.75 billion k-mers occupying 457.62 GB) (74). Before any calculations were made, the entire database was loaded into the NVRAM. With this configuration, the observed LMAT sustained sequence classification rate was 20 kbp/s/core. Finally, it is worth mentioning that a complete set of Python scripts was developed as back and front ends of the LMAT pipeline to manage the added complexity of time series analysis (https://github.com/DLSteam/MAUS_scripts).

### Taxon level robustness.

We selected the genus as the taxonomic level for the subsequent steps of our work. To ensure that there were no crucial differences between adjacent taxonomic levels that could still be of relevance after standardization (see the last subsection of Materials and Methods), we tested two different data sets. In the former, the antibiotic study (49) using 16S rRNA data, we tested the differences between the genus and family levels. The last data set tested was the kwashiorkor discordant-twin study (51) for both genus and species taxonomic levels. See Fig. S4 (overview) and S5 (detail) for plots comparing the studies (and so 16S rRNA and SMS data) and adjacent taxonomic levels.

10.1128/mSystems.00144-16.5 FIG S5  Detail of the comparison of different approaches based on adjacent taxonomic levels using plots of *x*-weighted power law fits (see Materials and Methods). The former row of subfigures shows examples for 16S rRNA, whereas the latter row of subfigures plots examples for SMS. The left column shows results for the superior taxonomic level (family for 16S rRNA, genus for SMS), while the right column shows results for the inferior level (genus for 16S rRNA, species for SMS). Download FIG S5, EPS file, 1.9 MB. Copyright © 2017 Martí et al. 2017 Martí et al. This content is distributed under the terms of the Creative Commons Attribution 4.0 International license.

### *x*-weighted power law fit.

When fitting the power law of standard versus mean, we took into account that every mean has uncertainty and can be estimated for a sample size *n* by the SEM (standard error of the mean). Here, the uncertainties affected the independent variable, so the fit was not as trivial as a *y*-weighted fit, where the uncertainties affect the dependent variable. A standard approach to perform this fit is to (i) invert the variables before applying the weights, (ii) perform the weighted fit, and (iii) revert the inversion. This method is deterministic, but the approximate solution worsens with smaller coefficients of determination. To overcome this limitation, we developed a stochastic method with a bootstrapping-like strategy that avoided inversion and was applicable regardless of the coefficient of determination.

The basic idea of bootstrapping is that inference about a population from sample data can be modeled by resampling the sample data and performing inference about a sample from resampled data (75). To adapt this general idea to our problem, we resampled the *x* data array by using its error array. That is, for each replicate, a new *x* data array was computed on the basis of $x_{i}^{*}=x_{i}+v_{i}$, where *v*_*i*_ is a Gaussian random variable with mean μ_*i*_ = 0 and standard deviation σ_*i*_ = SEM_*i*_, as defined previously. For each replicate, a complete unweighted power law fit was performed, where to choose between fitting power laws (*y* = *V*·*x*^β^) by linear regression on log-transformed data versus nonlinear regression, we mainly followed the general guidelines for the analysis of biological power laws (76). The parameters of the *x*-weighted fit were then estimated by averaging through all of the replicate fits performed, and their errors were estimated by computing the standard deviation for all of the fits. At the end of each step, the relative error was calculated by comparing the fit parameter estimation in the last step with the previous one. Finally, both the coefficient of determination of the fit and the coefficient of correlation between the fit parameters were estimated by averaging.

### RSI and variability.

The RSI is shown as a percentage in a separate bar on the right of the rank matrix plot in Fig. 4 and 5 (see also Fig. S1 and S2). The RSI is strictly 1 for an element whose range never changes over time and is strictly 0 for an element whose rank oscillates between the extremes from time to time. So, the RSI is calculated, per element, as 1 minus the quotient of the number of true rank hops taken divided by the number of maximum possible rank hops, all to the *p* power:

$$RSI={\left(1-\frac{\text{true}\;\text{rank}\;\text{hops}}{\text{possible}\;\text{rank}\;\text{hops}}\right)}^{p}={\left(1-\frac{D}{(N-1)\left(t-1\right)}\right)}^{p}$$

where *D* is the total number of rank hops taken by the element studied, *N* is the number of elements that have been ranked, and *t* is the number of time samples. The power index, *p* = 4, was arbitrarily chosen to increase the resolution in the stable region.

Finally, under the rank matrices in Fig. 4 and 5, there are plots relevant to the variability of the rank over time. On the one hand, the RV (rank variability) of a sampling point shows the absolute difference between every taxon’s rank and its accumulated abundance rank (the overall rank), averaged for all of the taxa shown. On the other hand, the DV (difference variability) of a sampling point shows the absolute difference between every taxon’s rank at that time and the value that it had at the previous sampling point, averaged for all of the taxa shown.

## References

[1] Rosenberg E, Zilber-Rosenberg I 2016 Microbes drive evolution of animals and plants: the hologenome concept. mBio 7:e01395-15. doi:10.1128/mBio.01395-15. PMC4817260 27034283

[2] Bordenstein SR, Theis KR 2015 Host biology in light of the microbiome: ten principles of holobionts and hologenomes. PLoS Biol 13:e1002226. doi:10.1371/journal.pbio.1002226. 26284777 PMC4540581

[3] Moran NA, Sloan DB 2015 The hologenome concept: helpful or hollow? PLoS Biol 13:e1002311. doi:10.1371/journal.pbio.1002311. 26636661 PMC4670207

[4] Swann JR, Want EJ, Geier FM, Spagou K, Wilson ID, Sidaway JE, Nicholson JK, Holmes E 2011 Systemic gut microbial modulation of bile acid metabolism in host tissue compartments. Proc Natl Acad Sci U S A 108:4523–4530. doi:10.1073/pnas.1006734107. 20837534 PMC3063584

[5] Spencer MD, Hamp TJ, Reid RW, Fischer LM, Zeisel SH, Fodor AA 2011 Association between composition of the human gastrointestinal microbiome and development of fatty liver with choline deficiency. Gastroenterology 140:976–986. doi:10.1053/j.gastro.2010.11.049. 21129376 PMC3049827

[6] Samuel BS, Shaito A, Motoike T, Rey FE, Backhed F, Manchester JK, Hammer RE, Williams SC, Crowley J, Yanagisawa M, Gordon JI 2008 Effects of the gut microbiota on host adiposity are modulated by the short-chain fatty-acid binding G protein-coupled receptor, Gpr41. Proc Natl Acad Sci U S A 105:16767–16772. doi:10.1073/pnas.0808567105. 18931303 PMC2569967

[7] Smith PM, Howitt MR, Panikov N, Michaud M, Gallini CA, Bohlooly-Y M, Glickman JN, Garrett WS 2013 The microbial metabolites, short-chain fatty acids, regulate colonic Treg cell homeostasis. Science 341:569–573. doi:10.1126/science.1241165. 23828891 PMC3807819

[8] Kimura I, Ozawa K, Inoue D, Imamura T, Kimura K, Maeda T, Terasawa K, Kashihara D, Hirano K, Tani T, Takahashi T, Miyauchi S, Shioi G, Inoue H, Tsujimoto G 2013 The gut microbiota suppresses insulin-mediated fat accumulation via the short-chain fatty acid receptor GPR43. Nat Commun 4:1829. doi:10.1038/ncomms2852. 23652017 PMC3674247

[9] Maslowski KM, Vieira AT, Ng A, Kranich J, Sierro F, Di Yu, Schilter HC, Rolph MS, Mackay F, Artis D, Xavier RJ, Teixeira MM, Mackay CR, Mackay CR 2009 Regulation of inflammatory responses by gut microbiota and chemoattractant receptor GPR43. Nature 461:1282–1286. doi:10.1038/nature08530. 19865172 PMC3256734

[10] Qin J, Li Y, Cai Z, Li S, Zhu J, Zhang F, Liang S, Zhang W, Guan Y, Shen D, Peng Y, Zhang D, Jie Z, Wu W, Qin Y, Xue W, Li J, Han L, Lu D, Wu P, Dai Y, Sun X, Li Z, Tang A, Zhong S, Li X, Chen W, Xu R, Wang M, Feng Q, Gong M, Yu J, Zhang Y, Zhang M, Hansen T, Sanchez G, Raes J, Falony G, Okuda S, Almeida M, LeChatelier E, Renault P, Pons N, Batto J-M, Zhang Z, Chen H, Yang R, Zheng W, Li S, Yang H, Wang J, Ehrlich SD, Nielsen R, Pedersen O, Kristiansen K, Wang J 2012 A metagenome-wide association study of gut microbiota in type 2 diabetes. Nature 490:55–60. doi:10.1038/nature11450. 23023125

[11] Brown JM, Hazen SL 2015 The gut microbial endocrine organ: bacterially derived signals driving cardiometabolic diseases. Annu Rev Med 66:343–359. doi:10.1146/annurev-med-060513-093205. 25587655 PMC4456003

[12] Durbán A, Abellán JJ, Jiménez-Hernández N, Artacho A, Garrigues V, Ortiz V, Ponce J, Latorre A, Moya A 2013 Instability of the faecal microbiota in diarrhoea-predominant irritable bowel syndrome. FEMS Microbiol Ecol 86:581–589. doi:10.1111/1574-6941.12184. 23889283

[13] Gevers D, Kugathasan S, Denson LA, Vázquez-Baeza Y, Van Treuren W, Ren B, Schwager E, Knights D, Song SJ, Yassour M, Morgan XC, Kostic AD, Luo C, González A, McDonald D, Haberman Y, Walters T, Baker S, Rosh J, Stephens M, Heyman M, Markowitz J, Baldassano R, Griffiths A, Sylvester F, Mack D, Kim S, Crandall W, Hyams J, Huttenhower C, Knight R, Xavier RJ 2014 The treatment-naive microbiome in new-onset Crohn’s disease. Cell Host Microbe 15:382–392. doi:10.1016/j.chom.2014.02.005. 24629344 PMC4059512

[14] Ridaura VK, Faith JJ, Rey FE, Cheng J, Duncan AE, Kau AL, Griffi NW, Lombard V, Henrissat B, Bain JR, Michael J, Ilkayeva O, Semenkovich CF, Funai K, Hayashi DK, Lyle BJ, Martini MC, Ursell LK, Clemente JC, Van Treuren W, William A, Knight R, Newgard CB, Heath AC, Gordon JI, Kau AL, Griffin NW, Muehlbauer MJ 2013 Gut microbiota from twins discordant for obesity modulate metabolism in mice. Science 341:1241214. doi:10.1126/science.1241214. PMC3829625 24009397

[15] Turnbaugh PJ, Hamady M, Yatsunenko T, Cantarel BL, Duncan A, Ley RE, Sogin ML, Jones WJ, Roe BA, Affourtit JP, Egholm M, Henrissat B, Heath AC, Knight R, Gordon JI 2009 A core gut microbiome in obese and lean twins. Nature 457:480–484. doi:10.1038/nature07540. 19043404 PMC2677729

[16] Subramanian S, Huq S, Yatsunenko T, Haque R, Mahfuz M, Alam MA, Benezra A, DeStefano J, Meier MF, Muegge BD, Barratt MJ, VanArendonk LG, Zhang Q, Province MA, Petri WA, Ahmed T, Gordon JI 2014 Persistent gut microbiota immaturity in malnourished Bangladeshi children. Nature 510:417–421. doi:10.1038/nature13421. 24896187 PMC4189846

[17] Moya A, Ferrer M 2016 Functional redundancy-induced stability of gut microbiota subjected to disturbance. Trends Microbiol 24:402–413. doi:10.1016/j.tim.2016.02.002. 26996765

[18] Cryan JF, Dinan TG 2012 Mind-altering microorganisms: the impact of the gut microbiota on brain and behaviour. Nat Rev Neurosci 13:701–712. doi:10.1038/nrn3346. 22968153

[19] Xu R, Wang Q 2016 Towards understanding brain-gut-microbiome connections in Alzheimer’s disease. BMC Syst Biol 10:63. doi:10.1186/s12918-016-0307-y. 27585440 PMC5009560

[20] Giloteaux L, Goodrich JK, Walters WA, Levine SM, Ley RE, Hanson MR 2016 Reduced diversity and altered composition of the gut microbiome in individuals with myalgic encephalomyelitis/chronic fatigue syndrome. Microbiome 4:30. doi:10.1186/s40168-016-0171-4. 27338587 PMC4918027

[21] Marchesi JR, Adams DH, Fava F, Hermes GDa, Hirschfield GM, Hold G, Quraishi MN, Kinross J, Smidt H, Tuohy KM, Thomas LV, Zoetendal EG, Hart A 2016 The gut microbiota and host health: a new clinical frontier. Gut 65:330–339. doi:10.1136/gutjnl-2015-309990. 26338727 PMC4752653

[22] Falony G, Joossens M, Vieira-Silva S, Wang J, Darzi Y, Faust K, Kurilshikov A, Bonder MJ, Valles-Colomer M, Vandeputte D, Tito RY, Chaffron S, Rymenans L, Verspecht C, De Sutter L, Lima-Mendez G, Dhoe K, Jonckheere K, Homola D, Garcia R, Tigchelaar EF, Eeckhaudt L, Fu J, Henckaerts L, Zhernakova A, Wijmenga C, Raes J 2016 Population-level analysis of gut microbiome variation. Science 352:560–564. doi:10.1126/science.aad3503. 27126039

[23] Zhernakova A, Kurilshikov A, Bonder MJ, Tigchelaar EF, Schirmer M, Vatanen T, Mujagic Z, Vila AV, Falony G, Vieira-Silva S, Wang J, Imhann F, Brandsma E, Jankipersadsing SA, Joossens M, Cenit MC, Deelen P, Swertz MA, Weersma RK, Feskens EJM, Netea MG, Gevers D, Jonkers D, Franke L, Aulchenko YS, Huttenhower C, Raes J, Hofker MH, Xavier RJ, Wijmenga C, Fu J 2016 Population-based metagenomics analysis reveals markers for gut microbiome composition and diversity. Science 352:565–569. doi:10.1126/science.aad3369. 27126040 PMC5240844

[24] Amato KR 2016 Incorporating the gut microbiota into models of human and non-human primate ecology and evolution. Am J Phys Anthropol 159:S196–S215. doi:10.1002/ajpa.22908. 26808106

[25] Wu H, Tremaroli V, Bäckhed F 2015 Linking microbiota to human diseases: a systems biology perspective. Trends Endocrinol Metab 26:758–770. doi:10.1016/j.tem.2015.09.011. 26555600

[26] Noecker C, Eng A, Srinivasan S, Theriot CM, Young VB, Jansson JK, Fredricks DN, Borenstein E 2016 Metabolic model-based integration of microbiome taxonomic and metabolomic profiles elucidates mechanistic links between ecological and metabolic variation. mSystems 1:e00013-15. doi:10.1128/mSystems.00013-15. PMC4883586 27239563

[27] Greenblum S, Turnbaugh PJ, Borenstein E 2012 Metagenomic systems biology of the human gut microbiome reveals topological shifts associated with obesity and inflammatory bowel disease. Proc Natl Acad Sci U S A 109:594–599. doi:10.1073/pnas.1116053109. 22184244 PMC3258644

[28] Bashan A, Gibson TE, Friedman J, Carey VJ, Weiss ST, Hohmann EL, Liu YY 2016 Universality of human microbial dynamics. Nature 534:259–262. doi:10.1038/nature18301. 27279224 PMC4902290

[29] Smith HF 1938 An empirical law describing heterogeneity in the yields of agricultural crops. J Agric Sci 28:1–23. doi:10.1017/S0021859600050516.

[30] Taylor LR 1961 Aggregation, variance and the mean. Nature 189:732–735. doi:10.1038/189732a0.

[31] de Menezes MA, Barabási AL 2004 Fluctuations in network dynamics. Phys Rev Lett 92:028701. doi:10.1103/PhysRevLett.92.028701. 14753972

[32] Mantegna RN, Stanley HE 1995 Scaling behaviour in the dynamics of an economic index. Nature 376:46–49. doi:10.1038/376046a0.

[33] Eisler Z, Kertész J, Yook S-H, Barabási A-L 2005 Multiscaling and non-universality in fluctuations of driven complex systems. Europhys Lett 69:664–670. doi:10.1209/epl/i2004-10384-1.

[34] Cohen JE, Xu M, Schuster WSF 2013 Stochastic multiplicative population growth predicts and interprets Taylor’s power law of fluctuation scaling. Proc Biol Sci 280:20122955. doi:10.1098/rspb.2012.2955. PMC3619479 23427171

[35] Reed DH, Hobbs GR 2004 The relationship between population size and temporal variability in population size. Anim Conserv 7:1–8. doi:10.1017/S1367943004003476.

[36] Anderson RM, Gordon DM, Crawley MJ, Hassell MP 1982 Variability in the abundance of animal and plant species. Nature 296:245–248. doi:10.1038/296245a0.

[37] Živkovíc J, Tadíc B, Wick N, Thurner S 2006 Statistical indicators of collective behavior and functional clusters in gene networks of yeast. Eur Phys J B 50:255–258. doi:10.1140/epjb/e2006-00103-4.

[38] Kendal WS 2003 An exponential dispersion model for the distribution of human single nucleotide polymorphisms. Mol Biol Evol 20:579–590. doi:10.1093/molbev/msg057. 12679541

[39] Zhang Z, Geng J, Tang X, Fan H, Xu J, Wen X, Ma ZS, Shi P 2014 Spatial heterogeneity and co-occurrence patterns of human mucosal-associated intestinal microbiota. ISME J 8:881–893. doi:10.1038/ismej.2013.185. 24132077 PMC3960530

[40] Kaltz O, Escobar-Páramo P, Hochberg ME, Cohen JE 2012 Bacterial microcosms obey Taylor’s law: effects of abiotic and biotic stress and genetics on mean and variance of population density. Ecol Process 1:5. doi:10.1186/2192-1709-1-5.

[41] Ramsayer J, Fellous S, Cohen JE, Hochberg ME 2012 Taylor’s law holds in experimental bacterial populations but competition does not influence the slope. Biol Lett 8:316–319. doi:10.1098/rsbl.2011.0895. 22072282 PMC3297404

[42] Pérez-Cobas AE, Artacho A, Ott SJ, Moya A, Gosalbes MJ, Latorre A 2014 Structural and functional changes in the gut microbiota associated to Clostridium difficile infection. Front Microbiol 5:335. doi:10.3389/fmicb.2014.00335. 25309515 PMC4163665

[43] Ding T, Schloss PD 2014 Dynamics and associations of microbial community types across the human body. Nature 509:357–360. doi:10.1038/nature13178. 24739969 PMC4139711

[44] Gajer P, Brotman RM, Bai G, Sakamoto J, Schütte UME, Zhong X, Koenig SSK, Fu L, Ma ZS, Zhou X, Abdo Z, Forney LJ, Ravel J 2012 Temporal dynamics of the human vaginal microbiota. Sci Transl Med 4:132ra52. doi:10.1126/scitranslmed.3003605. PMC3722878 22553250

[45] Blumm N, Ghoshal G, Forró Z, Schich M, Bianconi G, Bouchaud JP, Barabási AL 2012 Dynamics of ranking processes in complex systems. Phys Rev Lett 109:128701. doi:10.1103/PhysRevLett.109.128701. 23005999

[46] Eisler Z, Bartos I, Kertész J 2008 Fluctuation scaling in complex systems: Taylor’s law and beyond. Adv Phys 57:89–142. doi:10.1080/00018730801893043.

[47] Hidalgo CA, Blumm N, Barabási AL, Christakis NA 2009 A dynamic network approach for the study of human phenotypes. PLoS Comput Biol 5. doi:10.1371/journal.pcbi.1000353. PMC2661364 19360091

[48] Caporaso JG, Lauber CL, Costello EK, Berg-Lyons D, Gonzalez A, Stombaugh J, Knights D, Gajer P, Ravel J, Fierer N, Gordon JI, Knight R 2011 Moving pictures of the human microbiome. Genome Biol 12:R50. doi:10.1186/gb-2011-12-5-r50. 21624126 PMC3271711

[49] Dethlefsen L, Relman DA 2011 Incomplete recovery and individualized responses of the human distal gut microbiota to repeated antibiotic perturbation. Proc Natl Acad Sci U S A 108:4554–4561. doi:10.1073/pnas.1000087107. 20847294 PMC3063582

[50] Faith JJ, Guruge JL, Charbonneau M, Subramanian S, Seedorf H, Goodman AL, Clemente JC, Knight R, Heath AC, Leibel RL, Rosenbaum M, Gordon JI 2013 The long-term stability of the human gut microbiota. Science 341:1237439. doi:10.1126/science.1237439. PMC3791589 23828941

[51] Smith MI, Yatsunenko T, Manary MJ, Trehan I, Mkakosya R, Cheng J, Kau AL, Rich SS, Concannon P, Mychaleckyj JC, Liu J, Houpt E, Li JV, Holmes E, Nicholson J, Knights D, Ursell LK, Knight R, Gordon JI 2013 Gut microbiomes of Malawian twin pairs discordant for kwashiorkor. Science 339:548–554. doi:10.1126/science.1229000. 23363771 PMC3667500

[52] David LA, Maurice CF, Carmody RN, Gootenberg DB, Button JE, Wolfe BE, Ling AV, Devlin AS, Varma Y, Fischbach MA, Biddinger SB, Dutton RJ, Turnbaugh PJ 2014 Diet rapidly and reproducibly alters the human gut microbiome. Nature 505:559–563. doi:10.1038/nature12820. 24336217 PMC3957428

[53] David LA, Materna AC, Friedman J, Campos-Baptista MI, Blackburn MC, Perrotta A, Erdman SE, Alm EJ 2014 Host lifestyle affects human microbiota on daily timescales. Genome Biol 15:R89. doi:10.1186/gb-2014-15-7-r89. 25146375 PMC4405912

[54] Jørgensen B, Martinez JR, Tsao M 1994 Asymptotic behaviour of the variance function. Scand J Stat 21:223–243.

[55] Fronczak A, Fronczak P 2010 Origins of Taylor’s power law for fluctuation scaling in complex systems. Phys Rev E 81(Pt 2):066112. doi:10.1103/PhysRevE.81.066112. 20866483

[56] Kendal WS, Jørgensen B 2011 Taylor’s power law and fluctuation scaling explained by a central-limit-like convergence. Phys Rev E 83:066115. doi:10.1103/PhysRevE.83.066115. 21797449

[57] Kendal WS, Jørgensen B 2011 Tweedie convergence: a mathematical basis for Taylor’s power law, 1/f noise, and multifractality. Phys Rev E 84:066120. doi:10.1103/PhysRevE.84.066120. 22304168

[58] Kilpatrick AM, Ives AR 2003 Species interactions can explain Taylor’s power law for ecological time series. Nature 422:65–68. doi:10.1038/nature01471. 12621433

[59] Ballantyne F IV, Kerkhoff AJ 2007 The observed range for temporal mean-variance scaling exponents can be explained by reproductive correlation. Oikos 116:174–180. doi:10.1111/j.2006.0030-1299.15383.x.

[60] Stein RR, Bucci V, Toussaint NC, Buffie CG, Rätsch G, Pamer EG, Sander C, Xavier JB 2013 Ecological modeling from time-series inference: insight into dynamics and stability of intestinal microbiota. PLoS Comput Biol 9:e1003388. doi:10.1371/journal.pcbi.1003388. 24348232 PMC3861043

[61] Fisher CK, Mehta P 2014 Identifying keystone species in the human gut microbiome from metagenomic timeseries using sparse linear regression. PLoS One 9:e102451. doi:10.1371/journal.pone.0102451. 25054627 PMC4108331

[62] Bucci V, Tzen B, Li N, Simmons M, Tanoue T, Bogart E, Deng L, Yeliseyev V, Delaney ML, Liu Q, Olle B, Stein RR, Honda K, Bry L, Gerber GK 2016 MDSINE: Microbial Dynamical Systems INference Engine for microbiome time-series analyses. Genome Biol 17:121. doi:10.1186/s13059-016-0980-6. 27259475 PMC4893271

[63] Koenig JE, Spor A, Scalfone N, Fricker AD, Stombaugh J, Knight R, Angenent LT, Ley RE 2011 Succession of microbial consortia in the developing infant gut microbiome. Proc Natl Acad Sci USA 108:4578–4585. doi:10.1073/pnas.1000081107. 20668239 PMC3063592

[64] Weber J 1956 Fluctuation dissipation theorem. Phys Rev 101:1620–1626. doi:10.1103/PhysRev.101.1620.

[65] Larsen PE, Dai Y 2015 Metabolome of human gut microbiome is predictive of host dysbiosis. Gigascience 4:42. doi:10.1186/s13742-015-0084-3. 26380076 PMC4570295

[66] Peters BA, Dominianni C, Shapiro JA, Church TR, Wu J, Miller G, Yuen E, Freiman H, Lustbader I, Salik J, Friedlander C, Hayes RB, Ahn J 2016 The gut microbiota in conventional and serrated precursors of colorectal cancer. Microbiome 4:69. doi:10.1186/s40168-016-0218-6. 28038683 PMC5203720

[67] Gardiner BJ, Tai AY, Kotsanas D, Francis MJ, Roberts SA, Ballard SA, Junckerstorff RK, Korman TM 2015 Clinical and microbiological characteristics of Eggerthella lenta bacteremia. J Clin Microbiol 53:626–635. doi:10.1128/JCM.02926-14. 25520446 PMC4298500

[68] Tikhonov M 2016 Community-level cohesion without cooperation. Elife 5:e15747. doi:10.7554/eLife.15747. 27310530 PMC4946899

[69] Einstein A 1905 Über die von der molekularkinetischen Theorie der Wärme geforderte Bewegung von in ruhenden Flüssigkeiten suspendierten Teilchen. Ann Phys 322:549–560. doi:10.1002/andp.19053220806.

[70] Caporaso JG, Kuczynski J, Stombaugh J, Bittinger K, Bushman FD, Costello EK, Fierer N, Peña AG, Goodrich JK, Gordon JI, Huttley GA, Kelley ST, Knights D, Koenig JE, Ley RE, Lozupone CA, Mcdonald D, Muegge BD, Pirrung M, Reeder J, Sevinsky JR, Turnbaugh PJ, Walters Wa, Widmann J, Yatsunenko T, Zaneveld J, Knight R 2010 QIIME allows analysis of high-throughput community sequencing data intensity normalization improves color calling in SOLiD sequencing. Nat Methods 7:335–336. (Letter.) 20383131 10.1038/nmeth.f.303 PMC3156573

[71] Ames SK, Hysom DA, Gardner SN, Lloyd GS, Gokhale MB, Allen JE 2013 Scalable metagenomic taxonomy classification using a reference genome database. Bioinformatics 29:2253–2260. doi:10.1093/bioinformatics/btt389. 23828782 PMC3753567

[72] Gordon A, Hannon GJ 2010 FASTX-Toolkit: FASTQ/A short reads pre-processing tools v0.0.13. http://hannonlab.cshl.edu/fastx_toolkit/. Accessed: 26 July 2016.

[73] Quast C, Pruesse E, Yilmaz P, Gerken J, Schweer T, Yarza P, Peplies J, Glöckner FO 2013 The SILVA ribosomal RNA gene database project: improved data processing and web-based tools. Nucleic Acids Res 41:D590–D596. doi:10.1093/nar/gks1219. 23193283 PMC3531112

[74] Ames SK, Gardner SN, Marti JM, Slezak TR, Gokhale MB, Allen JE 2015 Using populations of human and microbial genomes for organism detection in metagenomes. Genome Res 25:1056–1067. doi:10.1101/gr.184879.114. 25926546 PMC4484388

[75] Wu CFJ 1986 Jackknife, bootstrap and other resampling methods in regression analysis. Ann Statist 14:1261–1295. doi:10.1214/aos/1176350142.

[76] Xiao X, White EP, Hooten MB, Durham SL 2011 On the use of log-transformation vs. nonlinear regression for analyzing biological power laws. Ecology 92:1887–1894. doi:10.1890/11-0538.1. 22073779

