# Supplementary material for: Health and Disease Imprinted in the Time Variability of the Human Microbiome
Source: mSystems. 2017 Mar 21;2(2):e00144-16. doi: 10.1128/mSystems.00144-16 (PMC5361781; doi:10.1128/mSystems.00144-16)
Supplement: TABLE S1 [file sys002172097st6.pdf]

Metadata	V	β	$\bar{R}^2$	V_{st}	β_{st}
A	0.26 ± 0.05	0.826 ± 0.025	0.918	3.1 ± 0.9	1.2 ± 0.6
A	0.32 ± 0.06	0.857 ± 0.025	0.924	4.4 ± 1.1	2.0 ± 0.6
A	0.194 ± 0.033	0.813 ± 0.024	0.918	1.9 ± 0.6	0.9 ± 0.6
A	0.24 ± 0.04	0.824 ± 0.020	0.924	2.7 ± 0.7	1.2 ± 0.5
A	0.34 ± 0.06	0.855 ± 0.024	0.931	4.7 ± 1.1	1.9 ± 0.6
A	0.30 ± 0.05	0.847 ± 0.022	0.921	3.9 ± 1.0	1.7 ± 0.5
A	0.133 ± 0.021	0.784 ± 0.023	0.916	0.7 ± 0.4	0.2 ± 0.6
A	0.25 ± 0.04	0.831 ± 0.024	0.929	3.0 ± 0.8	1.4 ± 0.6
P	0.23 ± 0.05	0.804 ± 0.035	0.885	2.6 ± 0.9	0.7 ± 0.8
P	0.097 ± 0.018	0.705 ± 0.031	0.891	0.03 ± 0.34	-1.6 ± 0.7
P	0.037 ± 0.006	0.642 ± 0.025	0.881	-1.12 ± 0.11	-3.1 ± 0.6
P	0.118 ± 0.019	0.723 ± 0.025	0.895	0.4 ± 0.4	-1.2 ± 0.6
P	0.17 ± 0.04	0.78 ± 0.04	0.842	1.5 ± 0.7	0.1 ± 0.9
P	0.123 ± 0.020	0.757 ± 0.026	0.914	0.5 ± 0.4	-0.4 ± 0.6
P	0.19 ± 0.05	0.77 ± 0.04	0.871	1.8 ± 0.9	-0.0 ± 0.9
P	0.121 ± 0.020	0.736 ± 0.027	0.921	0.5 ± 0.4	-0.9 ± 0.6
P	0.187 ± 0.034	0.771 ± 0.030	0.908	1.8 ± 0.7	-0.1 ± 0.7
P	0.097 ± 0.015	0.735 ± 0.025	0.922	0.05 ± 0.28	-0.9 ± 0.6
