# Supplementary material for: Health and Disease Imprinted in the Time Variability of the Human Microbiome
Source: mSystems. 2017 Mar 21;2(2):e00144-16. doi: 10.1128/mSystems.00144-16 (PMC5361781; doi:10.1128/mSystems.00144-16)
Supplement: TABLE S2 [file sys002172097st7.pdf]

Metadata	V	β	$\bar{R}^2$	V_{st}	β_{st}
Ab	0.35 ± 0.07	0.81 ± 0.04	0.925	4.3 ± 1.4	1.3 ± 0.9
Ab	0.41 ± 0.09	0.82 ± 0.04	0.908	5.6 ± 1.8	1.6 ± 0.9
Ab	0.23 ± 0.04	0.770 ± 0.031	0.920	2.1 ± 0.8	0.5 ± 0.7
Ab	0.165 ± 0.029	0.738 ± 0.031	0.928	0.9 ± 0.6	-0.3 ± 0.7
Ab	0.34 ± 0.06	0.812 ± 0.032	0.936	4.1 ± 1.2	1.5 ± 0.7
Ab	0.26 ± 0.05	0.798 ± 0.033	0.931	2.8 ± 0.9	1.1 ± 0.8
IBS (minor)	0.205 ± 0.034	0.740 ± 0.029	0.917	6.9 ± 3.3	2.0 ± 1.2
IBS (severe)	0.35 ± 0.06	0.793 ± 0.025	0.934	21 ± 6	4.2 ± 1.0
HLS (abroad)	0.51 ± 0.06	0.820 ± 0.012	0.928	2.8 ± 0.6	1.7 ± 0.5
HLS (infection)	0.49 ± 0.08	0.828 ± 0.018	0.923	2.6 ± 0.9	2.0 ± 0.7
HLS (after infection)	0.36 ± 0.05	0.776 ± 0.015	0.922	1.1 ± 0.6	-0.0 ± 0.6
