# Supplementary material for: Health and Disease Imprinted in the Time Variability of the Human Microbiome
Source: mSystems. 2017 Mar 21;2(2):e00144-16. doi: 10.1128/mSystems.00144-16 (PMC5361781; doi:10.1128/mSystems.00144-16)
Supplement: TABLE S3 [file sys002172097st8.pdf]

Metadata	V	β	$\bar{R}^2$	V_{st}	β_{st}
DH	0.27 ± 0.04	0.835 ± 0.016	0.925	0.2 ± 0.4	-1.0 ± 0.6
DH	0.36 ± 0.06	0.858 ± 0.015	0.929	1.1 ± 0.6	-0.2 ± 0.5
DH	0.35 ± 0.06	0.859 ± 0.014	0.926	1.0 ± 0.5	-0.1 ± 0.5
DH	0.25 ± 0.04	0.829 ± 0.014	0.911	0.0 ± 0.4	-1.2 ± 0.5
DH	0.30 ± 0.05	0.844 ± 0.014	0.920	0.5 ± 0.4	-0.7 ± 0.5
DH	0.29 ± 0.05	0.850 ± 0.016	0.915	0.4 ± 0.5	-0.5 ± 0.5
DH	0.28 ± 0.05	0.848 ± 0.016	0.921	0.3 ± 0.5	-0.5 ± 0.6
DH	0.35 ± 0.07	0.861 ± 0.017	0.918	0.9 ± 0.6	-0.0 ± 0.6
DH	0.31 ± 0.04	0.833 ± 0.012	0.916	0.6 ± 0.4	-1.1 ± 0.4
DH	0.33 ± 0.05	0.843 ± 0.013	0.925	0.8 ± 0.5	-0.7 ± 0.5
DH	0.31 ± 0.05	0.852 ± 0.014	0.925	0.6 ± 0.5	-0.4 ± 0.5
DH	0.31 ± 0.05	0.853 ± 0.015	0.930	0.6 ± 0.5	-0.4 ± 0.5
DH	0.203 ± 0.033	0.815 ± 0.015	0.907	-0.44 ± 0.32	-1.7 ± 0.5
DK	0.40 ± 0.07	0.859 ± 0.017	0.926	1.5 ± 0.7	-0.1 ± 0.6
DK	0.44 ± 0.08	0.868 ± 0.016	0.919	1.8 ± 0.8	0.2 ± 0.6
DK	0.196 ± 0.031	0.819 ± 0.014	0.916	-0.50 ± 0.30	-1.5 ± 0.5
DK	0.160 ± 0.026	0.798 ± 0.015	0.904	-0.85 ± 0.25	-2.3 ± 0.5
DK	0.30 ± 0.05	0.845 ± 0.014	0.924	0.5 ± 0.4	-0.6 ± 0.5
DK	0.23 ± 0.04	0.834 ± 0.014	0.908	-0.1 ± 0.4	-1.0 ± 0.5
DK	0.27 ± 0.05	0.848 ± 0.015	0.930	0.2 ± 0.4	-0.5 ± 0.5
DK	0.35 ± 0.07	0.860 ± 0.019	0.916	1.0 ± 0.7	-0.1 ± 0.7
DK	0.34 ± 0.05	0.835 ± 0.012	0.917	0.9 ± 0.5	-1.0 ± 0.4
DK	0.25 ± 0.04	0.831 ± 0.012	0.912	0.0 ± 0.4	-1.1 ± 0.4
DK	0.36 ± 0.06	0.858 ± 0.013	0.918	1.1 ± 0.5	-0.2 ± 0.5
DK	0.31 ± 0.06	0.851 ± 0.016	0.924	0.6 ± 0.6	-0.4 ± 0.6
DK	0.149 ± 0.022	0.799 ± 0.013	0.905	-0.96 ± 0.22	-2.2 ± 0.5
