# Supplementary material for: Health and Disease Imprinted in the Time Variability of the Human Microbiome
Source: mSystems. 2017 Mar 21;2(2):e00144-16. doi: 10.1128/mSystems.00144-16 (PMC5361781; doi:10.1128/mSystems.00144-16)
Supplement: TABLE S4 [file sys002172097st9.pdf]

Metadata	V	β	$\bar{R}^2$	V_{st}	β_{st}
OW	0.59 ± 0.12	0.894 ± 0.034	0.920	6.6 ± 2.0	2.6 ± 1.0
OW	0.22 ± 0.04	0.830 ± 0.030	0.904	0.5 ± 0.6	0.7 ± 0.9
OBI	0.28 ± 0.04	0.855 ± 0.022	0.958	1.5 ± 0.6	1.4 ± 0.6
OBI	0.33 ± 0.07	0.870 ± 0.031	0.916	2.4 ± 1.1	1.9 ± 0.9
OBII	0.223 ± 0.032	0.823 ± 0.023	0.938	0.6 ± 0.5	0.5 ± 0.7
OBII	0.208 ± 0.029	0.844 ± 0.022	0.935	0.4 ± 0.5	1.1 ± 0.7
OBIII	0.34 ± 0.05	0.855 ± 0.025	0.943	2.5 ± 0.9	1.4 ± 0.7
OBIII	0.26 ± 0.04	0.845 ± 0.026	0.954	1.1 ± 0.7	1.2 ± 0.8
OBIII	0.33 ± 0.06	0.870 ± 0.027	0.908	2.4 ± 1.0	1.9 ± 0.8
OBIII	0.200 ± 0.026	0.843 ± 0.020	0.949	0.2 ± 0.4	1.1 ± 0.6
OBIII	0.30 ± 0.05	0.846 ± 0.026	0.929	1.9 ± 0.8	1.2 ± 0.7
OBIII	0.176 ± 0.029	0.826 ± 0.026	0.894	-0.2 ± 0.5	0.6 ± 0.8
OBIII	0.30 ± 0.06	0.841 ± 0.031	0.896	1.8 ± 0.9	1.0 ± 0.9
OBIII	0.28 ± 0.04	0.857 ± 0.025	0.941	1.5 ± 0.7	1.5 ± 0.7
OBIII	0.122 ± 0.018	0.822 ± 0.024	0.930	-1.05 ± 0.30	0.5 ± 0.7
OBIIId	0.47 ± 0.08	0.872 ± 0.023	0.945	4.7 ± 1.3	1.9 ± 0.7
OBIIId	0.38 ± 0.06	0.846 ± 0.023	0.951	3.2 ± 1.0	1.2 ± 0.7
OBIIId	0.36 ± 0.06	0.842 ± 0.022	0.954	2.9 ± 0.9	1.1 ± 0.6
