# Supplementary material for: Health and Disease Imprinted in the Time Variability of the Human Microbiome
Source: mSystems. 2017 Mar 21;2(2):e00144-16. doi: 10.1128/mSystems.00144-16 (PMC5361781; doi:10.1128/mSystems.00144-16)
Supplement: TABLE S5 [file sys002172097st10.pdf]

Period		Genera											
		Actinomyces		***Leuconostoc***		***Lachnobacterium***		***Eggerthella***		***Clostridium***		***Collinsella***	
name	days	rank	RSI	rank	RSI	rank	RSI	rank	RSI	rank	RSI	rank	RSI
before	0 to 70	46	72.5	44	76.3	45	70.2	35	73.3	28	77.2	25	84.2
abroad	72 to 122	56	67.1	46	66.2	77	53.3	48	53.4	36	49.9	41	63.5
returned	123 to 256	44	79.3	41	69.5	31	74.2	33	77.5	34	71.6	27	81.0
after	257 to 364	43	79.0	39	72.2	33	68.4	30	78.5	34	76.7	26	80.4
Overall		47	76.4	43	71.0	36	69.2	35	74.1	34	70.7	28	79.5
